# COVID-19 in Patient with Sarcoidosis Receiving Long-Term Hydroxychloroquine Treatment, France, 2020

**DOI:** 10.3201/eid2610.201816

**Published:** 2020-10

**Authors:** François Bénézit, Audrey Le Bot, Stéphane Jouneau, Florian Lemaître, Charlotte Pronier, Pierre-Axel Lentz, Solène Patrat-Delon, Matthieu Revest, Vincent Thibault, Pierre Tattevin

**Affiliations:** Pontchaillou University Hospital, Rennes, France

**Keywords:** COVID-19, 2019 novel coronavirus disease, hydroxychloroquine, plasma concentration, sarcoidosis, France, severe acute respiratory syndrome coronavirus 2, SARS-CoV-2, coronavirus, coronavirus disease, respiratory infections, viruses, zoonoses

## Abstract

Because of in vitro studies, hydroxychloroquine has been evaluated as a preexposure or postexposure prophylaxis for coronavirus disease (COVID-19) and as a possible COVID-19 curative treatment. We report a case of COVID-19 in a patient with sarcoidosis who was receiving long-term hydroxychloroquine treatment and contracted COVID-19 despite adequate plasma concentrations.

Because of in vitro studies suggesting potential activity on severe acute respiratory syndrome coronavirus 2 (SARS-CoV-2) (*1*,*2*), hydroxychloroquine has been one of the main candidate drugs evaluated for coronavirus disease (COVID-19), both as a curative treatment and as preexposure or postexposure prophylaxis. We report a case of COVID-19 in a patient receiving long-term hydroxychloroquine treatment despite plasma concentrations within the therapeutic range for autoimmune diseases, such as systemic lupus erythematosus.

A 40-year-old man was admitted to Pontchaillou University Hospital, Rennes, France, for treatment of COVID-19. His medical history was remarkable only for pulmonary sarcoidosis, diagnosed in 2015; it was well controlled with hydroxychloroquine (200 mg 2×/d) with no other immunomodulatory drugs and no adherence issues. Twelve days before admission, he had received a diagnosis of COVID-19 in the outpatient department after a 4-day course of cough, myalgia, and low-grade fever. He had positive results by PCR for SARS-CoV-2 on a nasopharyngeal sample (RdRp gene; Pasteur COV_IP2/4, Paris, France; https://www.who.int/docs/default-source/coronaviruse/real-time-rt-pcr-assays-for-the-detection-of-sars-cov-2-institut-pasteur-paris.pdf?sfvrsn=3662fcb6_2). Physical examination was unremarkable except for a body temperature of 37.8°C. He was not admitted to the hospital at that time and was advised to continue his long-term treatment with hydroxychloroquine. His symptoms initially improved, but he developed shortness of breath with minimal exertion starting on day 14 of symptoms, gradually worsening over the next 2 days. He was admitted on day 16 because of constant shortness of breath and thoracic pain.

At admission, the patient’s body temperature was 36.6°C, heart rate was 82 beats/min, respiratory rate 20 breaths/min, blood pressure 115/72 mm Hg, and arterial oxygen saturation 96% while breathing room air. Lung auscultation revealed diffuse, fine crackles. Trough hydroxycholoroquine plasma concentration was 0.9 μg/mL (therapeutic range for autoimmune diseases 0.3–1.0 μg/mL). Thoracic computed tomography (CT) scan with pulmonary angiography ruled out pulmonary embolism but revealed diffuse ground-glass opacities, superimposed on the baseline sarcoidosis lesions (Figure). Electrocardiogram and serum troponin level were unremarkable. He was treated with prophylactic enoxaparin (60 mg 1×/d) and was discharged on day 18. Because he was afebrile and his condition improved shortly after admission, no additional workup for secondary pneumonia was performed, and he received no antibacterial treatment. All symptoms finally resolved, except for minor asthenia and cough (last follow-up at 40 days after discharge).

**Figure:**
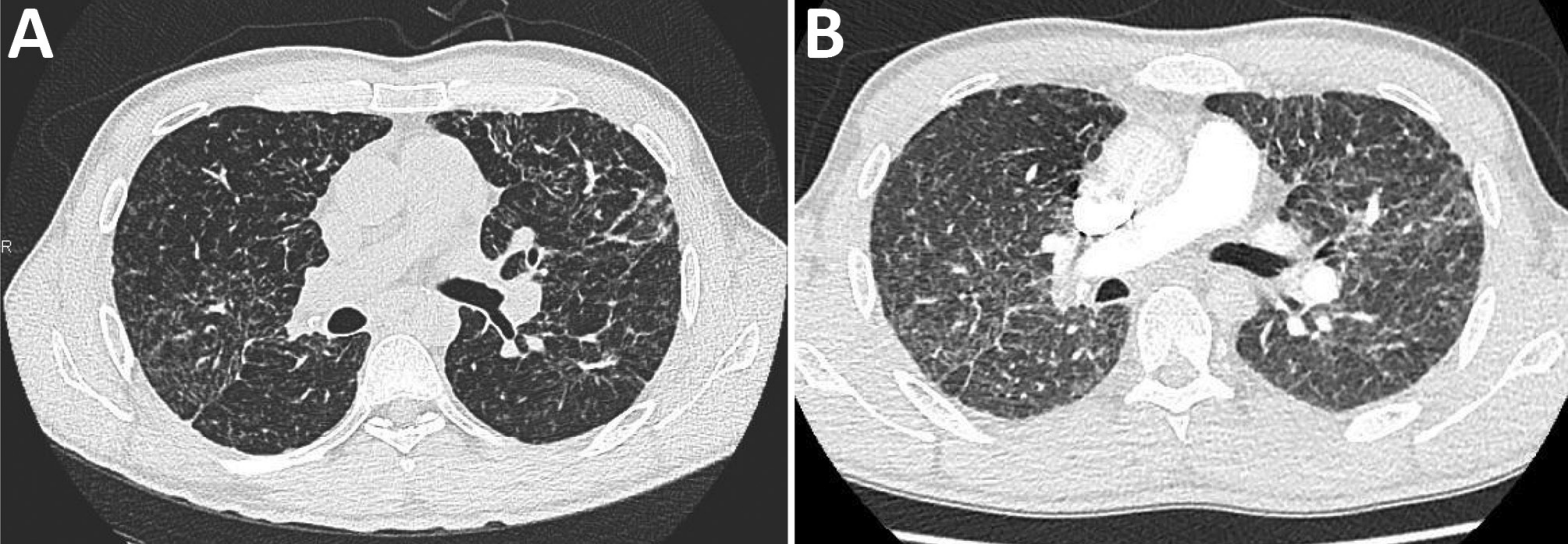
Computed tomography (CT) scans of a coronavirus disease (COVID-19) patient with sarcoidosis who had been receiving long-term hydroxychloroquine treatment, France. A) Thoracic CT scan from November 2019, showing baseline pulmonary sarcoidosis lesions. B) Thoracic CT scan performed April 4, 2020, showing diffuse ground-glass opacities characteristic of COVID-19.

This observation of COVID-19 with diffuse interstitial pneumonia requiring hospital admission in a patient on long-term hydroxychloroquine treatment suggests that hydroxychloroquine may not be as effective as suggested by in vitro data. This patient had always been considered highly adherent to his medications, which was confirmed by therapeutic drug monitoring. Because plasma concentration was within therapeutic range by the time the patient was admitted, the failure of hydroxychloroquine to prevent COVID-19 cannot be attributed to underdosage or suboptimal adherence. Two recent studies suggested that hydroxychloroquine provides no protection against COVID-19 in patients with a broad range of autoimmune diseases from New York, USA (*3*), and in patients with systemic lupus erythematosus from France (*4*). The case we present is unique in that the patient was not receiving any immunomodulatory agent other than hydroxychloroquine.

Our observations have limitations. First, no CT scan was performed during the first visit, and no nasopharyngeal PCR was performed at the second visit. However, this patient was managed in line with the recommendations in France and most other countries by that time. For the first visit, CT scan was not indicated because the diagnosis was obtained otherwise (positive PCR), and the patient had no criteria for admission; at the second visit, there was no indication to repeat PCR because it would have had no effect on the diagnosis or the management of the patient, and access to these tests was restricted. Second, the optimal dosing of hydroxychloroquine has not been defined for COVID-19; recent reports have suggested that target plasma concentrations should be 1–2 μg/mL in this population, based on chloroquine or hydroxychloroquine concentrations required to observe the virustatic effect in vitro and in silico (0.3–2.1 μg/mL) and toxic concentrations in humans (starting from 2 µg/mL) (*1**,**5*). Thus, the hydroxychloroquine plasma therapeutic range for autoimmune diseases may not be appropriate for the treatment of COVID-19: a dosage of 400 mg twice daily for 1 day, followed by 200 mg twice daily for another 4 days, has been recommended based on pharmacokinetic/pharmacodynamic data (*1*). Third, plasma concentration within the therapeutic range does not ensure that therapeutic concentrations are obtained in the lungs, the primary target for SARS-CoV-2.

Previous studies on hydroxychloroquine use during COVID-19 have found contradictory results, but they were all limited by small sample size, heterogenous hydroxychloroquine dosages, no or limited therapeutic drug monitoring, or methodological flaws (*6*). Ongoing randomized trials should resolve the ongoing controversy.

## References

[1] Yao X, Ye F, Zhang M, Cui C, Huang B, Niu P, et al. In vitro antiviral activity and projection of optimized dosing design of hydroxychloroquine for the treatment of severe acute respiratory syndrome coronavirus 2 (SARS-CoV-2). Clin Infect Dis. 2020 Mar 9 [Epub ahead of print]. 10.1093/cid/ciaa237 PMC7108130 32150618

[2] Liu J, Cao R, Xu M, Wang X, Zhang H, Hu H, et al. Hydroxychloroquine, a less toxic derivative of chloroquine, is effective in inhibiting SARS-CoV-2 infection in vitro. Cell Discov. 2020;6:16. 10.1038/s41421-020-0156-0 32194981 PMC7078228

[3] Haberman R, Axelrad J, Chen A, Castillo R, Yan D, Izmirly P, et al. Covid-19 in Immune-Mediated Inflammatory Diseases—Case Series from New York. N Engl J Med. 2020 Apr 29 [Epub ahead of print]. 10.1056/NEJMc2009567 PMC7204427 32348641

[4] Mathian A, Mahevas M, Rohmer J, Roumier M, Cohen-Aubart F, Amador-Borrero B, et al. Clinical course of coronavirus disease 2019 (COVID-19) in a series of 17 patients with systemic lupus erythematosus under long-term treatment with hydroxychloroquine. Ann Rheum Dis. 2020 Apr 24 [Epub ahead of print]. 10.1136/annrheumdis-2020-217566 32332072

[5] Perinel S, Launay M, Botelho-Nevers É, Diconne É, Louf-Durier A, Lachand R, et al. Towards optimization of hydroxychloroquine dosing in intensive care unit COVID-19 patients. Clin Infect Dis. 2020 Apr 7 [Epub ahead of print]. 10.1093/cid/ciaa394 PMC7184449 32255489

[6] Yazdany J, Kim AHJ. Use of hydroxychloroquine and chloroquine during the COVID-19 pandemic: what every clinician should know. Ann Intern Med. 2020 Mar 31 [Epub ahead of print]. 10.7326/M20-1334 PMC7138336 32232419

